# Catastrophic health expenditure in households with chronic disease patients: A pre-post comparison of the New Health Care Reform in Shaanxi Province, China

**DOI:** 10.1371/journal.pone.0194539

**Published:** 2018-03-16

**Authors:** Yongjian Xu, Jie Ma, Na Wu, Xiaojing Fan, Tao Zhang, Zhongliang Zhou, Jianmin Gao, Jianping Ren, Gang Chen

**Affiliations:** 1 School of Public Policy and Administration, Xi’an Jiaotong University, Xi’an, China; 2 Jinhe Center for Economic Research, Xi’an Jiaotong University, Xi’an, China; 3 Department of Human Resources, Xi’an Central Hospital, Xi’an, China; 4 School of Public Health, Health Science Center, Xi’an Jiaotong University, Xi’an, China; 5 School of Medicine, Hangzhou Normal University, Hangzhou, China; 6 Centre for Health Economics, Monash Business School, Monash University, Melbourne, Australia; Centre Hospitalier Universitaire Vaudois, FRANCE

## Abstract

**Introduction:**

In 2009, China officially launched the New Health Care Reform (NHCR). One important purpose of the reform was to reduce financial burden of health care through health insurance expansion and health care provider regulations. This study aimed to provide evidence on the effect of the NHCR reform on catastrophic health expenditure (CHE) by comparing the occurrence and inequality of CHE among households with chronic diseases patients before and after the reform.

**Methods:**

This study used the subset of data from the 2008 and 2013 National Health Services Survey conducted in Shaanxi Province. Our sample included households with chronic diseases patients and excluded observations with key variables missing. The final sample size was 1942 households in 2008 and 7704 households in 2013. We defined CHE occurrence following the definition of the World Health Organization (WHO). The income-related inequality in CHE was measured by the concentration index. A multi-level logistic regression model was used in the study to explore the influence of the NHCR on CHE occurrence, controlling for important covariates.

**Results:**

From 2008 to 2013, the occurrence rate of CHE in rural areas declined from 29.15% to 23.62%. However, the CHE rate in urban areas increased from 19.18% to 24.95%. The interaction term between year and rural/urban location was statistically significant, confirming that the influence of the NHCR on the CHE occurrence rates were heterogeneous between rural and urban areas. As for the CHE inequality, the concentration index in rural areas decreased from -0.4572 to -0.5499 with a p-value less than 0.05. This implied that the CHE occurrence inequality was increased after the implementation of the NHCR.

**Conclusion:**

Our study suggested that the implementation of the NHCR might not have been effective in reducing the CHE occurrence for households with chronic disease patients. Although the occurrence of CHE of rural households had decreased, the occurrence of CHE in urban areas was higher than before. In addition, the income inequality of CHE occurrence was greater in 2013 compared to that in 2008 in rural areas. Although the reform resulted in higher insurance coverage and higher government expenditure in health care, the financial burden of health care on households did not necessarily improve. Further efforts on developing the current health insurance system and optimizing the hierarchical health care system are required to improve the protection against CHE.

## Introduction

With the development of industrialization and urbanization, the accelerated pace of population aging, and the increasing practice of unhealthy lifestyles, the prevalence of chronic diseases including diabetes, hypertension, and some types of cancers in China has been increasing rapidly in recent years [1–2]. The National Health and Family Planning Commission of China estimated that chronic diseases had become the leading cause of death and disability in China, responsible for 86.6% of all deaths in 2015[3].

The growing prevalence of chronic diseases also contributed to high financial risks of health care faced by households in China. The total out-of-pocket (OOP) payments as a proportion of total healthcare expenditure had increased rapidly from 20% in the late 1970s before China’s economic reform to 55% in 1998, and remained high despite substantial efforts to improve health care affordability including launching the New Cooperative Medical Scheme in 2003. In 2008, the OOP payments still made up about 40% of total health care expenditure [4]. The OOP expenditure of chronic diseases was estimated to account for nearly 70% of the total OOP expenditure [3].

Extremely high OOP health expenditure relative to income can result in financial catastrophe. Catastrophic health expenditure (CHE) is used to describe the level of health expenditure that poses a threat to financial capacity and capability of household to maintain household basic subsistence [5]. The World Health Organization (WHO) defines CHE as OOP greater than or equal to 40% of a household’s capacity to pay [6]. Households with CHE must cut down expenditure on necessities, such as food, clothing and education. In extreme cases, some households sink into a vicious circle of poverty and ill health.

In 2009, China officially launched the New Health Care Reform (NHCR). One of the main purposes of this reform was to reduce the financial burden of health care on households. The NHCR was a comprehensive reform consisting of health insurance expansion and structural changes on health care providers. The NHCR aimed to develop a universal health insurance system covering both urban and rural residents. By 2013, 95.1% of residents in China were enrolled in one of the three types of basic health insurance schemes: Urban Employee Basic Medical Insurance (UEBMI) for urban employees, the Urban Resident Basic Medical Insurance (URBMI) for urban residents without formal employment, and the New Rural Cooperative Medical Insurance (NRCMI) for rural residents [7]. In rural areas, the reform also added specific chronic disease subsidies to help pay for treatments of a set of chronic diseases [8].

The health care provider reform aimed to establish a national essential-medicine system [9]. All primary care facilities that received specific subsidies from government were required to provide and distribute a catalogue of basic prescription drugs at zero markup. This requirement substantially reduced the price of basic prescription drugs faced by patients seeking care in primary care facilities [10]. Other efforts included promoting a set of free basic public health services such as check-ups and chronic disease screening for elderly residents. However, despite these efforts, data of National Health and Family Planning Commission of China showed that OOP still accounted for more than 31% of total health expenditure in 2014 [11].

Most previous empirical studies of the CHE in China measured the occurrence of CHE and identified determinants using cross-sectional data of one period [12–13]. Very few studies have compared the occurrence of CHE before and after the NHCR using repeated cross-sectional data. This study provided evidence on the influence of the NHCR on CHE occurrence. Our study had two objectives: one was to describe the occurrence and inequality of CHE before and after the implementation of NHCR; the other was to explore the potential influence of the NHCR on CHE using multivariate multilevel models.

We used the subsample of the 2008 and 2013 National Health Services Survey conducted in Shaanxi Province. The analysis employed a multi-level logistic regression to account for the hierarchical sampling structure used to collect the data. Compared to models without the multi-level structure used in most previous studies, our model could capture the influence of community-level factors and improve the estimation of standard errors. We focused on households with chronic diseases patients because these households faced higher financial burdens and were more likely to experience CHE. In 2008, households with chronic diseases patents were 10.32% more likely to endure CHE compared to the average household.

This study contributed to the literature in two ways. Firstly, the repeated cross-sectional data allowed us to estimate the effect of the NHCR on the occurrence of CHE, providing one of the first policy evaluation of whether the NHCR had helped to reduce financial risks of health care as one primary objective of the reform. Understanding the influence of the NHCR on CHE is important because although the primary goal of the reform was to reduce financial burdens of health care, there had been concerns that it had failed to do so. Secondly, the rich information in the data set allowed us to explore how the NHCR’s effects interacted with other important determinants of the CHE. This could help us not only understand the heterogeneity of the effects, but also the possible mechanisms of the impact. By discussing the heterogeneity of the effects on households, for example by different economic backgrounds, the evidence may point to new directions for future health care reform.

## Methods

### Data source

The data used in this study was obtained from the 2008 and 2013 National Health Services Survey (NHSS) conducted in Shaanxi Province. The NHSS was conducted by the Center for Health Statistics and Information under the National Health and Family Planning Commission. The survey was designed primarily to provide information on health care utilization, health care payment, and trends in health and health behaviors. Interviews were conducted in person by qualified investigators. More details on investigation contents and quality assurance measures can be found in previous studies [14–15]. This study used information from the household health survey component.

The NHSS adopted a four-stage stratified random cluster sampling method at county (district), township or neighborhood, village or neighborhood committees, and household level. The survey in Shaanxi province selected 5960 households in 2008 and 19127 households in 2013. This study focused on households with at least one member diagnosed with chronic diseases including diabetes, hypertension and so on (hereafter referred to as chronic households). We further excluded households with missing or anomalous expenditure data (medical and other types) from the analysis. Our final sample size was 1942 chronic households in 2008 and 7704 in 2013.

### Ethical considerations

The National Bureau of Statistics of China approved the procedures for the fourth and fifth NHSS data collection (license number (2008)18 and 2013(65), respectively). Informed consent was obtained from household members before the interview. Approval for this study was obtained by the Ethics Committee of Health Science Center, Xi’an Jiaotong University (approval number 2015–644).

### Statistical analysis

CHE occurs when the OOP payment for health care consumes a dangerously large portion of a household’s disposable income such that the household may be pushed into poverty. The consensus among economists and epidemiologists is that a household is in catastrophe if its OOP health payment exceeds a critical threshold [16]. The WHO proposed in 2005 that the threshold to be the OOP payment for health care exceeding 40% of the household's capacity to pay (CTP, defined as income remaining after subsistence needs have been met) [17].

We followed the WHO definition of the CHE in this study. A dummy variable indicating the occurrence of the CHE took value 1 if the fraction of OOP divided by CTP was larger than or equal to 0.4, and 0 otherwise. OOP was the total out-of-pocket expenditure on health services in a year; if the household received reimbursement from third-parties such as a private insurer or the government after the service, the amount was deducted in the calculation. CTP was computed as below:

$$CTP=\left\{ \begin{array}{c} exp-se\;if\;se\leq food\\ exp-food\;if\;se>food \end{array}\right.$$

where *exp* indicated the total household expenditure; *se* represented the household subsistence expenditure; *food* was the expenditure on food. We estimated the subsistence expenditure per capita as a weighted average of per person food expenditure of households in the 45–55 percentile in terms of food expenditure. *se* used in the calculation was the product of the subsistence expenditure and the household size. The reason to use the higher one between *se* and *food* for calculating the CTP was because the household’s own food expenditure might already be suffering from the pressure of high OOP.

We used a multivariate multi-level logistic regression model to explore the influence of the NHCR on CHE occurrence. Previous studies mostly used classic regression models[5, 12]. However, data used in these studies generally processed a hierarchical or clustered structure because of a multi-stage stratified sampling process used in data collection and patterns of health outcomes being correlated within a small geographic location. For example, households from the same town might share similar preference for health care, face similar constraints from the provider side, and be influenced by similar environmental factors. Ignoring the correlation within clusters can lead to underestimating standard errors and thus increase the risk of drawing incorrect statistically significant conclusions. The multilevel models took into account the correlation within clusters and could improve estimation of coefficients as well as standard errors [18].

We first checked if there existed a multi-level structure in the data by estimating a logistic model without any covariates but fixed and random effects at the group level. The model used was shown below:

$$\mathrm{logit}(P_{is})=\beta_{0}+u_{0s}+\varepsilon_{is}$$

Here *β*_0_ was the fixed intercept across all groups and *u*_0*s*_ was the random effect of each group (or the residual at the group level). The variance of *u*_0_ was the random coefficient which represents the level of group clustering. When $\sigma_{u_{0}}^{2}$ approached 0, the model became a single-level logistic regression. Thus, $\sigma_{u_{0}}^{2}$ could be used to test for group clustering. We estimated the model with group effects at the county, town, and village level and found statistical significance in variances at the county and town levels. Therefore, we chose a three-level logistic regression model to estimate the influence of the NHCR on CHE. The model specification was shown below

$$\mathrm{logit}\left(P_{ijk}\right)=\beta_{0}+\beta_{1}{post}_{ijk}+\beta_{3}{post}_{k}*{rural}_{ijk}+\beta_{4}{rural}_{ijk}+X_{ijk}\gamma +Z_{ijk}\delta +u_{0j}+v_{0k}+\varepsilon_{jik}$$

where *post*_*ijk*_ was the dummy variable representing the post reform period of 2013; *rural*_*ijk*_ was a dummy variable that took value 1 if the household was residing in rural areas and 0 if in urban areas. We included an interaction term between *post*_*ijk*_ and *rural*_*ijk*_ to capture the different changes between urban and rural areas. The reform’s health insurance expansion was focused on urban areas from 2008 to 2013 so the effect would differ between the rural and urban areas. *X*_*ijk*_ was the vector of household characteristics and *Z*_*ijk*_ a vector of household head’s characteristics such as age, gender, etc. Here *u*_0*j*_ and *v*_0*k*_ were the residuals for county and town levels.

Household characteristics included household economic status, household size (1, 2–4, ≥5), whether there were elderly members (≥60-year-old) or children under 5-year-old in the household, and whether obtained commercial insurance. Household economic status was measured by net per capita household expenditure which is total household expenditure minus health expenditure last year. Households were ranked by an ascending order of per-capital net household expenditure within rural and urban areas and grouped equally into five quintiles. The poorest 20% of households were grouped into the poorest group and the wealthiest 20% the richest group. Characteristics of the household head were also included in our analysis: age (<45, 45–60, ≥60), gender, education (illiteracy, elementary, middle school, high school and above), marital status (never married, married, others), and employment status of the household head.

Concentration curve and concentration index are commonly used to measure the degree of income-related inequality in the distribution of a health variable [19–20]. The concentration curve plots the cumulative percentage of the health variable on the y-axis against the cumulative percentage of the sample ranked by income from the poorest to the richest on the x-axis. The 45-degree line represents the line of equality where everyone has the same value of the health variable regardless of income. The further the curve is below the line of equality, the more concentrated the health variable is amongst the richer. The concentration curve can be used to compare the inequality visually. Concentration index is calculated based on the area between the line of equality and the concentration curve. The concentration index is between -1 and 1. A positive number indicates that the health variable is distributed toward the rich [21]. In the analysis, the health variable of interest is the occurrence of CHE.

All of analysis were conducted using Stata software version 14.0.

## Results

We first reported summary statistics of the household and the household head’s characteristics in 2008 and 2013 in Table 1. There were some notable differences between 2008 and 2013. Higher percentage of households were from rural areas in 2013 due to sampling structure changes. A much higher proportion of household heads was employed in 2013. These differences suggested that it was important to control for household characteristics in the analyses.

**Table 1:** Characteristics of chronic households and household heads.

	*2008*		*2013*	
	*n*	*%*	*n*	*%*
Location				
*Urban*	954	49.12	2870	37.25
*Rural*	988	50.88	4834	62.75
Commercial insurance				
*Yes*	247	12.72	740	9.61
*No*	1695	87.28	6964	90.39
Household size				
*1*	199	10.25	835	10.84
*2–4*	1436	73.94	5777	74.99
*≥5*	307	15.81	1092	14.17
Having elderly members				
*Yes*	1238	63.75	4418	57.35
*No*	704	36.25	3286	42.65
Having children				
*Yes*	1692	87.13	6619	85.92
*No*	250	12.87	1085	14.08
Household economic status				
*Quintile 1(poorest)*	390	20.08	1541	20.00
*Quintile 2(poorer)*	389	20.03	1545	20.05
*Quintile 3(middle)*	387	19.93	1537	19.95
*Quintile 4(richer)*	388	19.98	1541	20.00
*Quintile 5(richest)*	388	19.98	1540	20.00
Age of household head				
*<45*	447	23.02	1405	18.24
*45–60*	822	42.33	3061	39.73
*≥60*	673	34.65	3238	42.03
Gender of household head				
*Male*	1397	71.94	5774	74.95
*Female*	545	28.00	1930	25.05
Education of household head				
*Illiteracy*	315	16.22	1228	15.94
*Elementary*	519	26.73	2285	29.66
*Middle school*	661	34.04	2832	36.76
*High school and above*	447	23.02	1359	17.64
Marital status of household head				
*Unmarried*	50	2.57	207	2.69
*Married*	1583	81.51	6547	84.98
*Others*	309	15.91	950	12.33
Employment status of household head				
*Yes*	1142	58.81	5496	71.34
*No*	800	41.19	2208	28.66

Note: Data used here was from the 2008 and 2013 National Health Services Survey (NHSS) conducted in Shaanxi Province. This table reported unweighted averages. Other marital status included widow, divorced and other scenarios.

Table 2 summarized the CHE occurrence among chronic households in rural and urban areas. The first observation was that the overall level of CHE occurrence remained stable from 2008 to 2013 at 24%. The level of CHE was higher than most comparable countries [22–23], echoing the problem of unaffordable medical care. However, although the overall CHE occurrence rate remained stable, CHE occurrence in rural areas dropped from 29.15% to 23.62%, while CHE in urban areas increased from 19.18% to 24.95%. Both were significant at 1% level. The pattern here was very striking: in 2008 rural areas had much higher CHE occurrence compared to that in urban areas, while in 2013 the rates were almost equivalent.

**Table 2:** CHE occurrence among chronic households.

	2008		2013		*χ*^2^	*P*
	n	%	n	%		
Urban	183	19.18	716	24.95	13.235	<0.001
Rural	288	29.15	1142	23.62	13.517	<0.001
Total	471	24.25	1858	24.12	0.016	0.900

Note: This table reported the number of households with CHE occurrence among chronic households in 2008 and 2013. The data used was the 2008 and 2013 National Health Services Survey (NHSS) conducted in Shaanxi Province.

Table 3 showed CHE occurrence by household economic status. Here households were stratified into 5 quantiles based on their income ranking within rural and urban areas, from the poorest to the richest. The occurrence of CHE was notably lower for more financially affluent households. Among urban households, the CHE occurrence appeared to be higher in 2013 compared to 2008, and the increase was significant among the poorest and the middle-income households. In contrast, rural households experienced a reduction in CHE occurrence, except for the poorest and richest households.

**Table 3:** CHE occurrence by economic status.

	2008		2013		*χ*^2^	*P*
	n	%	n	%		
Urban						
*Quintile 1(poorest)*	79	41.36	318	55.21	11.015	0.044
*Quintile 2(poorer)*	54	28.27	168	29.37	0.084	0.772
*Quintile 3(middle)*	27	14.14	141	24.52	9.032	0.003
*Quintile 4(richer)*	14	7.33	67	11.69	2.877	0.090
*Quintile 5(richest)*	9	4.74	22	3.83	0.299	0.584
Rural						
*Quintile 1(poorest)*	107	53.77	538	55.64	0.232	0.629
*Quintile 2(poorer)*	76	38.58	276	28.54	7.815	0.005
*Quintile 3(middle)*	53	26.90	168	17.37	9.664	0.002
*Quintile 4(richer)*	39	19.70	114	11.79	9.009	0.003
*Quintile 5(richest)*	13	6.60	46	4.76	1.147	0.284

Note: This table reported the CHE occurrence in chronic households in 2008 and 2013, by income groups. The data used was the 2008 and 2013 National Health Services Survey (NHSS) conducted in Shaanxi Province.

Table 4 presented estimates from the three-level logistic regression. The interaction term between post and rural was negative and significant at 5% level, confirming observations in Table 3 that the reform was associated with a reduction in the CHE occurrence in rural areas compared to that in urban. Several other observations are also worth noting. Firstly, larger households had lower risk of CHE occurrence. Secondly, having elderly members in the household was associated with an increase in CHE occurrence, which was in line with that fact older people tend to have higher medial risks. Thirdly, comparing to the poorest households, higher income unsurprisingly shielded households from CHE occurrence, consistent with the observation in Table 3. The household head being employed was also associated with lower CHE. Forth, having household heads older than 45 was associated with higher CHE occurrence. This would have to do with both lower earning ability and higher medical risk. Education at the high school level or higher reduced CHE occurrence. Lastly and interestingly, having commercial health insurance seemed to be also associated with an increase in CHE, which could be reflecting the possibility of adverse selection in the commercial insurance market. The coefficient was not significant at the conventional level (p-value = 0.154) so we cannot say for sure, but the possibility may be worth further exploration.

**Table 4:** Multilevel random intercept logistic regression.

	Coef.	*Std*. *Err*.	*z*	*P>z*	*95% CI*	
					Low	High
Post	0.24	0.168	1.44	0.149	-0.087	0.573
Rural	-0.05	0.206	-0.26	0.794	-0.457	0.349
Post*Rural	-0.49	0.229	-2.16	0.031	-0.943	-0.045
Commercial insurance	0.16	0.113	1.43	0.154	-0.060	0.383
Household size						
*1*	Ref					
*2–4*	-0.65	0.104	-6.19	<0.001	-0.850	-0.441
*≥5*	-1.20	0.142	-8.51	<0.001	-1.482	-0.927
Having elderly members	0.15	0.068	2.17	0.030	0.014	0.283
Having children	0.15	0.095	1.55	0.122	-0.039	0.332
Economic status						
*Quintile 1(poorest)*	Ref					
*Quintile 2(poorer)*	-0.93	0.077	-12.09	<0.001	-1.079	-0.778
*Quintile 3(middle)*	-1.62	0.088	-18.35	<0.001	-1.788	-1.443
*Quintile 4(richer)*	-2.13	0.098	-21.74	<0.001	-2.321	-1.937
*Quintile 5(richest)*	-3.44	0.136	-25.28	<0.001	-3.710	-3.176
Age of household head						
*<45 years*	Ref					
*45–60 years*	0.27	0.092	2.91	0.004	0.088	0.450
*≥60 years*	0.70	0.097	7.22	<0.001	0.512	0.893
Female Household head	0.14	0.073	1.87	0.062	-0.007	0.280
Education of household head						
*Illiteracy*	Ref					
*Elementary*	-0.04	0.083	-0.50	0.615	-0.204	0.121
*Middle school*	-0.14	0.090	-1.54	0.123	-0.316	0.038
*High school and above*	-0.26	0.112	-2.35	0.019	-0.483	-0.044
Marital status of household head						
*Never married*	Ref					
*Married*	0.11	0.173	0.66	0.511	-0.225	0.452
*Others*	-0.27	0.188	-1.45	0.146	-0.640	0.095
Household head employed	-0.41	0.077	-5.30	<0.001	-0.555	-0.255
Intercept	0.20	0.367	0.55	0.582	-0.518	0.922
Random-effects						
*Level 3*: *county*	0.09	0.041			0.034	0.218
*Level 2*: *town*	0.26	0.049			0.182	0.378

Note: This table reported the results from the multi-level logistic regression. The dependent variable is the CHE occurrence, and the independent variables are listed in the first column. The data used was the 2008 and 2013 National Health Services Survey (NHSS) conducted in Shaanxi Province.

**Fig 1:**
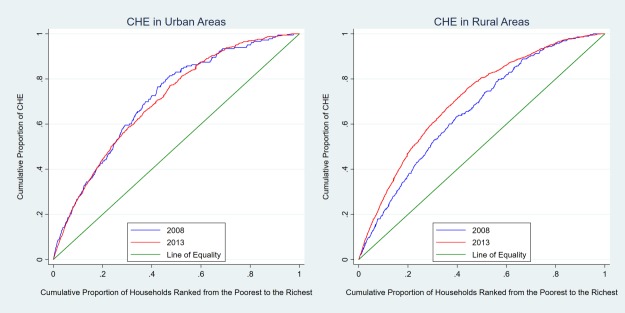
Concentration curves of CHE occurrence in urban and rural areas. The right and left plots show the distribution of CHE occurrence by household economic status in urban and rural areas, respectively. The green line in both plots are the equality line. The blue line and red line in both plots above the equality line represent the concentration curve in 2008 and 2013, respectively. The further the concentration curve is above the equality line, the more concentrated the catastrophic health expenditure is among poor households.

Next, we examined the distribution of CHE occurrence by income. Fig 1 plotted the concentration curves of the CHE occurrence by year in rural and urban areas. In both graphs the concentration curves were above the line of equality, indicating that CHE was more likely to occur among poorer households. While the two lines in the left panel (urban areas) were mostly overlapping, the 2013 line in the right panel was notably further away from the line of equality. This suggested that the CHE occurrence was skewed further toward poorer households in rural areas in 2013 compared to 2008. Although CHE occurrence in the rural area was lower after the reform, the benefit was more concentrated on relatively affluent households. The reform was not the most helpful for those poor households in rural areas which would be in the most need of protection against CHE.

Table 5 reported the concentration index of CHE occurrence by year in rural and urban areas. The numbers confirm the pattern in Fig 1: the concentration index decreased from -0.4572 to -0.5499 in rural areas and the change was significant at 5% level, suggesting an increase in inequality of CHE distribution.

**Table 5:** Concentration indices of CHE in 2008 and 2013.

	2008		2013		*Z*	*P*
	Normalized CI	Std. error	Normalized CI	Std. error		
Urban	-0.5255	0.0444	-0.5444	0.0227	-0.38	0.7055
Rural	*-0*.*4572*	*0*.*0377*	*-0*.*5499*	*0*.*0179*	*-2*.*22*	*0*.*0264*
Total	*-0*.*5049*	*0*.*0284*	*-0*.*5220*	*0*.*0142*	*-0*.*54*	*0*.*5902*

Note: This table reported the concentration indices in 2008 and 2013 in urban and rural areas.

## Discussion

Chinese government launched the NHCR in 2009 and one important aim was to mitigate the financial burdens of health care. This study provided new evidence on the effectiveness of the reform on reducing CHE occurrence among households with chronic diseases patients, using data from the 2008 and 2013 National Health Services Survey conducted Shaanxi Province. The evidence suggested that the occurrence rate of CHE in 2013 remained high around 24% and was not significantly different from 2008.

One study compared 14 countries in Asia and found that higher CHE was correlated with higher OOP [24]. Past studies also suggested that health care system which mainly relied on OOP had poor performance both in health care outcomes as well as health equality [25–27]. Only when OOP payed for less than 30% of total health care expenditure, the occurrence of CHE could be mostly eliminated [17]. However, the OOP share in Shaanxi Province was 38.84% in 2013 while government spending paid for only 28.93% [28]. With high OOP, it was also possible that patients would forego timely treatment and end up using less efficient and more expensive acute treatment with worse health outcomes. This situation was especially severe among chronic diseases patients who require constant management. The high OOP would incur high financial burdens among these households and even lead to poverty.

Our finding suggested that the reform was associated with a reduction in CHE occurrence from 2008 to 2013 in rural areas compared to urban areas. There were several factors that might contribute to the different responses in rural and urban areas. Firstly, the health care utilization increased faster in urban areas. In 2013, the proportion used hospital inpatient care was 10.47% in urban areas, increasing by 5.42 percentage points compared to 2008. In contrast, the hospital inpatient care rate increased by only 4.09 percentage points in rural areas.

Secondly, the urban residents were more likely to seek care in higher-tier hospitals: 27.5% went to city or province level hospitals to receive inpatient service in 2013 compared to 13.22% among rural residents. Similar services cost more in higher-tier hospitals so the medical expenses would be higher.

Another important provision of the reform was to mandate zero-profit in prescription drug pricing for local lower-tiered medical centers. Since the rural residents were more likely to use health care provided by local medical centers, they would be more likely to benefit from the price reduction in prescription drugs as well.

Lastly, the reform might have different provisions in rural and urban areas as well. The New Rural Cooperative Medical Insurance (NRCMI) which provided health insurance for rural population was the least generous among the three basic health insurance forms in 2008 [29]. However, the NHCR implemented comprehensive reimbursement policies for chronic diseases under NRCMI, while reimbursement policies for chronic diseases under urban insurances had changed little before and after the NHCR.

We also examined the distribution of CHE using concentration curve and index. CHE was more likely to occur among poorer households in both years. The inequality was not reduced from 2008 to 2013; the poorest quantile of urban households even experienced an increase in CHE occurrence from 2008 to 2013. In rural areas, although the occurrence of CHE was reduced, the inequality increased as the CHE reduction was concentrated among middle income households but not the poorest households. It appeared that the reform had not helped those who needed help in health care financing the most.

Based on our findings, there are a few policy implications on reducing CHE occurrence, especially among the poorest households. Despite the near universal coverage of basic health care, the extent of protection is limited by annual imbursement limits, incomplete catalog of covered services and drugs, and so on. The current health insurance system still leaves too much financial risk for households to bear, and thus the high occurrence of CHE [13]. Further reform is required to continue expanding the coverage of health insurance and increasing government spending on health care. In the design of basic health insurance, providing extra protection against costly chronic health conditions would be helpful.

Another aspect of efficient health care utilization requires directing patients away from seeking care directly at high-tier hospitals. This has long been a problem that patients would not go to primary care facilities and being treated or transferred from there but make high-tier hospitals the first stop. This practice resulted in wasted resources at primary care facilities and overly crowded high-tier hospitals which are much more expensive for treating minor diseases. One important reason is the low quality of health services at primary care facilities. One study showed that 64% of the prescribed drugs by local doctors may be harmful [30]. To improve the quality of local health centers would be an effective way to properly distribute patients and lower health care costs. In addition, the current fee-for-service health care financing is likely to lead to over utilization of health care and unnecessary financial risk. The payment system would need to pivot toward a pay-for-performance approach to improve cost effectiveness in health care.

The reduction of CHE in rural chronic households compared to urban households also suggests that the chronic diseases coverage might be very effective in reducing CHE among chronic households. The two urban insurances may consider providing special provision for chronic diseases reimbursement. China is in the process of combining the rural residents’ health insurance and urban residents’ health insurance: the new combined insurance may also consider retaining the design of the chronic diseases coverage.

The evidence that the CHE reduction being concentrated among middle income households but not the poorest households suggests that additional help is needed to improve the financial standing of the poor facing health care cost. One possible way is to provide additional coverage for low-income households: lower copayment, higher reimbursement rate and additional coverage beyond the reimbursement cap of general insurance. In addition, the health coverage may be combined with the delivery of cash assistance program in China for low-income households facing CHE.

This study had some limitations. Firstly, the repeated cross-sectional structure of our data and the nature of the reform limited our ability to draw causal conclusions of the impact of the reform. Since the reform affected everyone in China simultaneously-it was implemented at the same time across the nation in 2009, it was difficult to construct a control group as well. The findings were interpreted as associations rather than causal impact. Second, the survey information was self-reported so subject to recall biases as well as nonrandom missing. Luckily the survey was conducted carefully with detailed explanation of data confidentiality prior to the interview.

In conclusion, the implementation of the reform may have helped reducing financial risks of health care in rural areas. However, the effect was more limited for urban households and the overall level of CHE occurrence remained high. Poorer households in rural areas also benefited less from the reform. Further reform of the health care system is required to reduce the financial risk of health care, especially among poor households.
